# A novel mode of action for COX‐2 inhibition: Targeting ATPase domain of HSP90 induces ubiquitin degradation of new client protein COX‐2

**DOI:** 10.1002/ctm2.705

**Published:** 2022-01-24

**Authors:** Man Zhang, Jing Cui, Fukui Shen, Lili Ye, Chuanjing Cheng, Yang Li, Qiuyang Zhang, Lin Niu, Yuanyuan Hou, Gang Bai

**Affiliations:** ^1^ State Key Laboratory of Medicinal Chemical Biology College of Pharmacy and Tianjin Key Laboratory of Molecular Drug Research Nankai University Tianjin People's Republic of China; ^2^ Thompson Rivers University Manna British Columbia Canada; ^3^ Tianjin University of Traditional Chinese Medicine Tianjin People's Republic of China

Dear Editor,

Cyclooxygenase 2 (COX‐2) is the main target of nonsteroidal anti‐inflammatory drugs (NSAIDs),^1^ and it is rapidly expressed in response to extracellular factor stimulation like lipopolysaccharide (LPS) in mouse monocyte macrophage 264.7 (RAW264.7) cells.^2^ Herein, we reveal the existence of a novel mechanism that intervenes the formation of the heat shock protein 90 (HSP90)/COX‐2 complex and induces the ubiquitin‐proteasomal degradation of COX‐2.

Baicalein, the aglycone of baicalin, is a key antipyretic component of the classic traditional Chinese medicinal herb *Scutellaria baicalensis* Georgi (Huangqin),^3^ showed better antipyretic effects and nitric oxide inhibition than baicalin (Figure 1A‐C). We synthesized and evaluated several molecular probes based on baicalein (Figure 1D, Figures S1‐S7), and the potential targets captured by baicalein probe modified magnetic microspheres were identified by sodium dodecyl sulfate‐polyacrylamide gel electrophoresis, protein profiling analysis, and cell co‐localization imaging (Figure 1E‐G). The results of these analyses showed that HSP90 was the most likely target of baicalein. HSP90, the most common chaperone protein, could help its client proteins to fold correctly to exert their biological activity.^4^ Then, two known client proteins of HSP90—JNK and AKT—were dephosphorylated by baicalein (Figure 1H).

**FIGURE 1:**
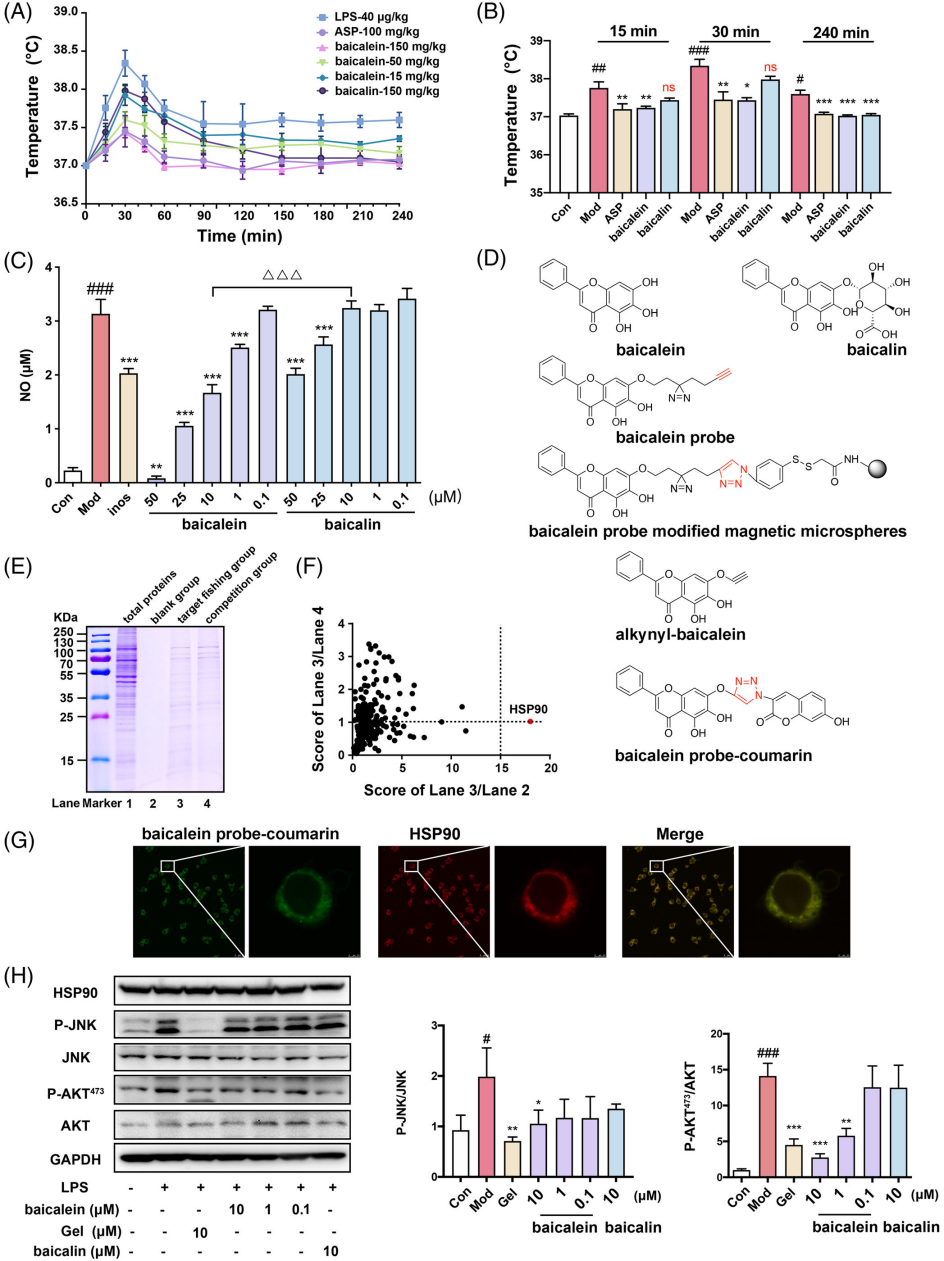
The antipyretic activity evaluation of baicalein, the target fishing, and co‐localization of baicalein with its target protein HSP90. (A and B) Comparison of the antipyretic activities of baicalein and baicalin in rats (15, 30, 240 min), (*n* = 5). (C) The effects of baicalein and baicalin on lipopolysaccharide (LPS)‐induced nitric oxide (NO) production (*n* = 3). (D) The structure of baicalein, baicalin, baicalein probe, baicalein probe modified magnetic microspheres, alkynyl‐baicalein and baicalein probe‐coumarin. (E) Potential target fishing of baicalein probe. Sodium dodecyl sulfate‐polyacrylamide gel electropheresis (SDS‐PAGE) was used to show the captured proteins. Lane 1 represented total protein from RAW264.7 cell lysate; lanes 2 and 3 represented proteins captured by blank magnetic microspheres or baicalein probe modified magnetic microspheres from RAW264.7 cell lysates, respectively; lane 4 represented proteins from the cells incubated with 5‐μM baicalein and 1‐μM baicalein probe, then captured by magnetic microspheres from RAW264.7 cell lysates. (F) Protein profiling analysis of captured proteins. HSP90 was revealed as the most likely target compared to other proteins, which had the highest score of lane 3/lane 2. (G) Colocalization of the baicalein probe‐coumarin (green) and HSP90 protein (red) in RAW264.7 cells. (H) Baicalein down‐regulated the protein expression levels of phosphorylation‐mitogen‐activated protein kinase 8 (P‐JNK) and P‐AKT^473^ stimulated by LPS (100 ng/mL). Bars represent the mean ± SD (*n* = 3). Significant differences between two groups were assessed using *t*‐tests, and analysis of multiple groups was performed using one‐way analysis of variance (ANOVA). ^#^ *p* < .05; ^##^ *p* < .01; ^###^ *p* < .001 compared with the Con group; ^*^ *p* < .05; ^**^ *p* < .01; ^***^ *p* < .001 compared with the Mod group. ^△△△^ *p* < .001 compared between two groups

The binding affinity between baicalein and HSP90, as detected by surface plasmon resonance (SPR), was approximately 26.07 μM (Figure 2A, Figure S8), while baicalin could not bind to HSP90 (Figure S9). The adenosine triphosphatase (ATPase) domain is considered the most important in HSP90. When HSP90 binds to adenosine‐triphosphate (ATP), transient dimerization occurs in the N‐terminal region, leading to HSP90 inactivation.^5^ The binding affinity between ATP and HSP90 was 0.6 mM, as detected by SPR; this affinity increased approximately six‐fold to 3.5 mM when baicalein competed with ATP to bind to HSP90 (Figure 2B). Geldanamycin (Gel), an HSP90 ATPase domain inhibitor, weakened the fluorescence resonance energy transfer of the baicalein probe‐coumarin/HSP90 (Figure 2C, Figure S10). Moreover, baicalein markedly inhibited the ATPase activity of HSP90 (Figure 2D).^6^

**FIGURE 2:**
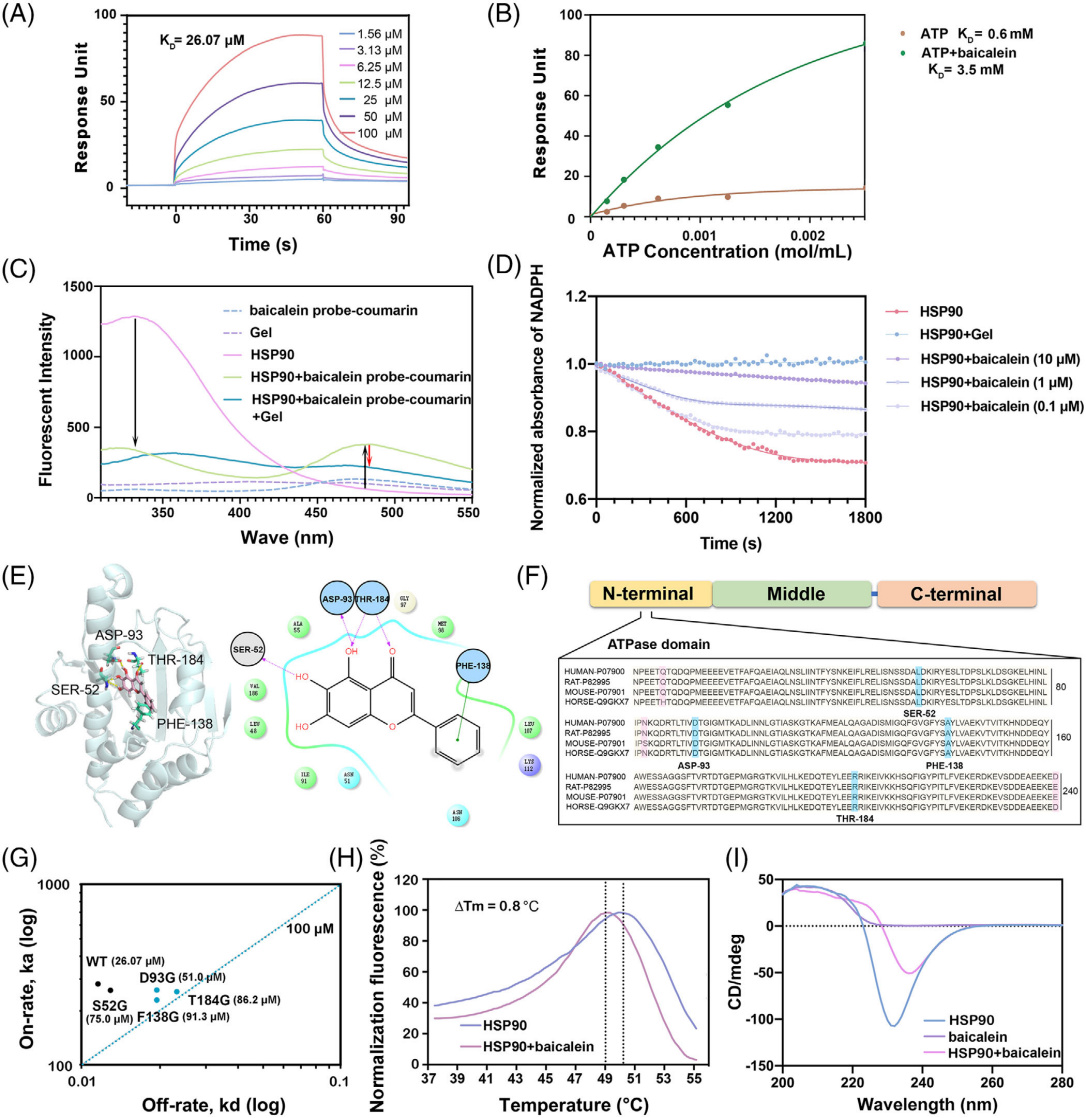
Interaction verification and site analysis between baicalein and HSP90 protein. (A) Surface plasmon resonance (SPR) analysis of interactions between baicalein and HSP90. A Biacore CM5 chip was used to capture HSP90. Measures of baicalein/HSP90 association and dissociation were performed in the presence of baicalein concentrations ranging from 1.56 to 100 μM. (B) SPR analysis of the competitive relationship between ATP and baicalein. (C) Fluorescence resonance energy transfer (FRET) measurements of baicalein probe‐coumarin and HSP90 proteins show an increase/decrease in acceptor/donor fluorescence at 475 and 290 nm, respectively. (D) Baicalein inhibited ATPase activity of HSP90 in a dose‐response manner. (E) The molecular docking of baicalein and HSP90 (1YET) by AutoDock (left panel) and MOE Dock (MOE) (right panel) software. *ΔG *= −8.19 kcal/mol. (F) Sequence alignment analysis of the ATPase domain of the HSP90 family in different species. (G) Amino acids SER52, ASP93, PHE138 and THR184 in the ATPase domain of HSP90 were turned to glycine as HSP90 mutants S52G, D93G, F138G and T184G. The on‐off rate of baicalein incubated with different HSP90 mutant proteins by SPR assay. (H) The thermal stability assay of the HSP90 protein with or without 10‐μM baicalein incubation assessed by fluorescence‐based thermal shift (FTS) assay. (I) The conformational state of the HSP90 protein (40 μM) analysed by circular dichroism spectroscopy (CD) assay after treatment with 10‐μM baicalein

Molecular docking studies illustrated that baicalein interacted with the amino acid residues—SER‐52, ASP‐93, PHE‐138, and THR‐184—in the ATPase domain of HSP90 with a binding energy of −8.19 kcal/mol (Figure 2E). These key amino acid sites are highly conserved in human, rat, mouse and horse species (Figure 2F).^7^, ^8^, ^9^ The on‐off image demonstrated that baicalein had a faster binding rate and slower dissociation rate than the mutants in the SPR assay (Figure 2G, Figures S11‐S14). In addition, baicalein caused a 0.8°C decrease in the protein melting temperature (*ΔT*) (Figure 2H). Circular dichroism spectroscopy revealed that the optical activity of HSP90 was partially changed after incubation with baicalein (Figure 2I). These results suggest that baicalein targets the ATP‐binding domain of HSP90.

A co‐immunoprecipitation (Co‐IP) test was designed to explore any unknown potential client proteins of HSP90 responsible for the antipyretic effects exerted by baicalein (Figure S15). The results of this test demonstrated that COX‐2, instead of COX‐1, could be captured by HSP90 in LPS‐stimulated RAW264.7 cells (Figure 3A). By integrating data on Co‐IP protein profiling (Table S1), client proteins of HSP90 and proteins related to inflammation and heat‐clearing from GeneCards, we constructed a Venn diagram, which suggested COX‐2 as a new client protein of HSP90 (Figure 3B). The binding affinity between COX‐2 and HSP90 was 1.17 μM, as detected by microscale thermophoresis. After pretreatment with baicalein, the specific binding aforementioned almost disappeared (Figure 3C, Figure S16).

**FIGURE 3:**
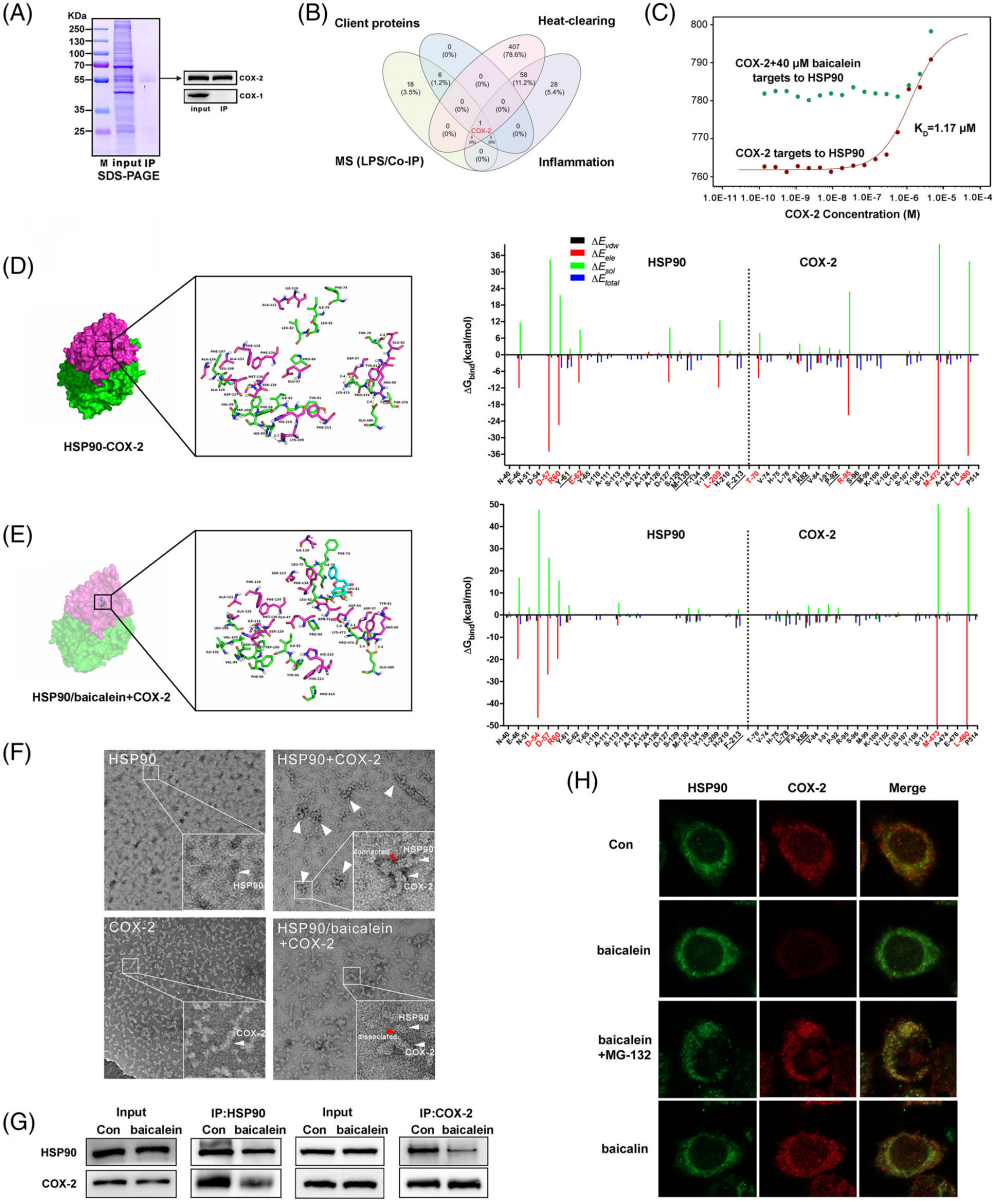
Baicalein inhibits the interaction of HSP90/COX‐2 and induces COX‐2 ubiquitin degradation, which was discovered and identified as a new client protein of HSP90. (A) SDS‐PAGE and western blot assay of the enriched proteins captured by HSP90 in co‐immunoprecipitation (Co‐IP) assay. (B) Venn diagram of the Co‐IP proteins and the proteins related to inflammation and heat‐clearing from GeneCards. (C) MicroScale thermophoresis (MST) analysis of COX‐2 and HSP90 proteins with or without baicalein. (D and E) The predicted binding modality of the HSP90/COX‐2 complex and HSP90/baicalein+COX‐2 complex obtained from molecular dynamics (MD) simulations. Residues contributing to *∆E*_ele_ were signed by red color, and residues contributing to *∆E*_vdw_ were marked by underline. (F) The protein interaction pattern observed in transmission electron microscope (TEM) images for HSP90, COX‐2 protein or HSP90+COX‐2 and HSP90/baicalein+COX‐2 protein complexes; the red arrow indicated the binding position of the two proteins. (G) Co‐IP of the HSP90 and COX‐2 proteins with or without baicalein. (H) Co‐localization of HSP90 proteins (green) and COX‐2 proteins (red). RAW264.7 cells were stimulated by lipopolysaccharide (LPS) with or without baicalein (10 μM), baicalein (10 μM) plus MG‐132 (10 μM) or baicalin (10 μM) for 24 h

Next, molecular docking analysis and molecular dynamics simulation were performed, which estimated the *∆G*_bind_ values of the HSP90/COX‐2 and HSP90/baicalein+COX‐2 complexes as −55.9 kcal/mol and −47.5 kcal/mol, respectively. The potential binding sites contributed to *∆E*_ele_ that weakened by baicalein were suggested as GLU‐62, LEU‐209 in HSP90 and THR‐70, ARG‐95 in COX‐2 (Figure 3D,E and Figures S17‐S19; Table S2). Transmission electron microscopy and Co‐IP results further demonstrated that baicalein promoted the dissociation of HSP90 and COX‐2 (Figure 3F,G). Since COX‐2 is a substrate of the ubiquitin‐proteasome system,^10^ the evidence of colocalization of HSP90 and COX‐2 revealed that baicalein instead of baicalin induced COX‐2 degradation, which could be reversed by the ubiquitin‐proteasome inhibitor MG‐132 (Figure 3H). Moreover, COX‐2 levels were decreased by baicalein administration (Figure S20).

Almost no normal COX‐2 protein was expressed in RAW264.7 cells, which were stimulated by LPS under baicalein treatment for 120 min, and the proteins sized approximately 35 kDa were predicted to be ubiquitin‐degraded COX‐2 proteins (Figure 4A). Consistent with this, several ubiquitin‐conjugates were formed following MG‐132 treatment (Figure 4B). As expected, the expression of COX‐1 and HSP90 at the protein level changed minimally (Figure 4C,D). The degradation of COX‐2 induced by baicalein almost disappeared when the cells were pretreated with HSP 90α/β siRNA (Figure S21). Hence, baicalein could decrease the expression of downstream effectors of COX‐2, including prostaglandin E2 (PGE2), interleukin‐6 (IL‐6) and tumor necrosis factor‐α (TNF‐α), in LPS‐stimulated RAW264.7 cells by inhibiting HSP90 in a manner similar to that of Gel (Figure 4E‐G).

**FIGURE 4:**
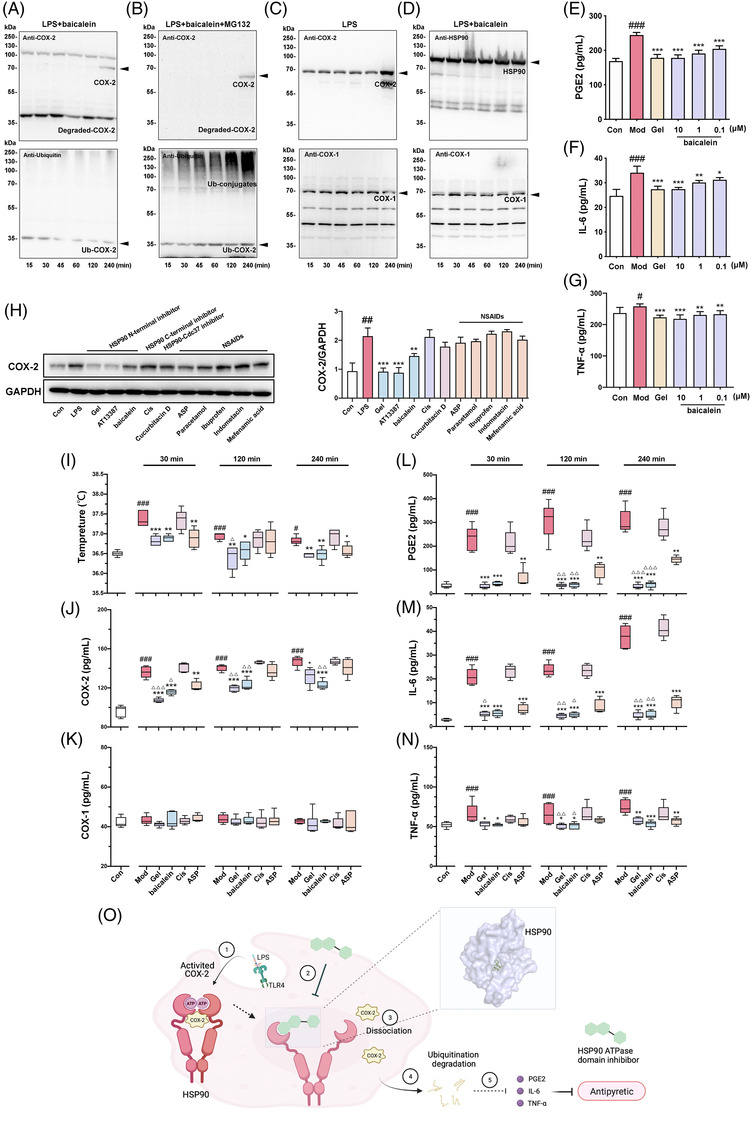
HSP90 ATPase domain inhibitors induce COX‐2 ubiquitin‐dependent degradation selectively via HSP90 and showed excellent antipyretic activity at different times. (A and B) COX‐2 ubiquitin degradation in lipopolysaccharide (LPS)‐stimulated RAW264.7 cells, detected by Anti‐COX‐2 or Anti‐Ubiquitin antibody, in the presence of baicalein or baicalein plus MG‐132. (C) The protein level of COX‐2 and COX‐1 under the treatment of LPS. (D) The protein level of HSP90 and COX‐1 in the presence of baicalein in LPS‐stimulated RAW264.7 cells. (E‐G) Baicalein inhibited the production of PGE2, IL‐6 and TNF‐α detected by enzyme‐linked immuno sorbent assay (ELISA), which induced by LPS in RAW264.7 cells (*n* = 6). Geldanamycin (Gel) (2.5 μM) was set as a positive control. (H) Effects of different kinds of HSP90 inhibitors and representative NSAIDs on COX‐2 protein expression stimulated by LPS for 4 h. Bars represent the mean ± SD (*n* = 3). (I) Comparison of the antipyretic activities of Gel, baicalein, cisplatin (Cis) and aspirin (ASP) in rats at 30, 120 and 240 min. (G) HSP90 ATPase domain inhibitors selectively induced COX‐2 proteins degradation in vivo. (K) Protein levels of COX‐1 in rats’ serum affected by LPS and with or without HSP90 inhibitors’ intervention. (L‐N) Baicalein inhibited the production of PGE2, IL‐6 and TNF‐α in febrile rats’ serum detected by ELISA (*n* = 5). (O) A schematic diagram illustrating the activation and degradation process of COX‐2 proteins accompanied with HSP90 and its ATPase inhibitor. Bars represent minimum to maximum in the boxplot. Statistical tests were calculated using GraphPad Prism software (GraphPad Software, La Jolla, CA, USA). Significant differences between two groups were assessed using *t*‐tests, and analysis of multiple groups was performed using one‐way ANOVA. Boxplot showing medians with 25th and 75th percentiles was made using GraphPad Prism 9. Comparison between the groups was performed using Mann–Whitney *U* test. ^#^ *p* < .05; ^##^ *p* < .01; ^###^ *p* < .001 compared with the Con group. ^*^ *p* < .05; ^**^ *p* < .01; ^***^ *p* < .01 compared with the Mod group. ^△^ *p* < .05; ^△△^ *p* < .01; ^△△△^ *p* < .01 compared with the ASP group simultaneously

Moreover, the effects of some known HSP90 inhibitors, such as Gel, AT13387, cisplatin (Cis), cucurbitacin D and five representative NSAIDs (aspirin [ASP], paracetamol, ibuprofen, indometacin and mefenamic acid), on COX‐2 degradation were compared. Only HSP90 ATPase domain inhibitors markedly decreased the expression of COX‐2 at the protein level (Figure 4H). In a rat model of pyrexia, the HSP90 ATPase domain inhibitors, Gel (1 mg/kg) and baicalein (1 mg/kg), exhibited significant antipyretic activity compared with Cis (1 mg/kg) and ASP (20 mg/kg) within 30 min. Interestingly, this effect lasted for 240 min (Figure 4I, Figure S22). Meanwhile, MG‐132 reduced the antipyretic effect of baicalein (Figure S23). HSP90 ATPase domain inhibitors inhibited the increase in COX‐2 levels induced by LPS more stably and durably than ASP (Figure 4J). However, there was no effect on COX‐1 levels under the same conditions (Figure 4K). Moreover, PGE2, IL‐6 and TNF‐α levels in the serum demonstrated the same effects at the cellular level (Figure 4L‐N).

In conclusion, COX‐2 was identified as a new client protein of HSP90. HSP90 ATPase domain inhibitors trigger the dissociation of the HSP90/COX‐2 complex and induce the degradation of COX‐2, which plays an antipyretic role (Figure 4O). Our findings demonstrate another mechanism for inhibiting COX‐2 and present a new strategy for the development of antipyretic and analgesic drugs.

## CONFLICT OF INTEREST

The authors have declared that no competing interest exists.

## Supporting information

Supporting Information

Supporting Information
