# Supplementary material for: A novel mode of action for COX‐2 inhibition: Targeting ATPase domain of HSP90 induces ubiquitin degradation of new client protein COX‐2
Source: Clin Transl Med. 2022 Jan 24;12(1):e705. doi: 10.1002/ctm2.705 (PMC8787097; doi:10.1002/ctm2.705)
Supplement: Supplementary file 1 — Supporting Information [file CTM2-12-e705-s002.docx]

**Supporting Information 1: Supplementary figures and tables**

**A novel mode of action for COX-2 inhibition: baicalein targets the ATPase domain of HSP90, inducing ubiquitin degradation of its newly identified client protein COX-2**

Man Zhang^1^, Jing Cui^1^, Fukui Shen^1^, Lili Ye^1^, Chuanjing Cheng^1^, Yang Li^1^, Qiuyang Zhang^2^, Lin Niu^3^, Yuanyuan Hou^1^*, Gang Bai^1^*

1 State Key Laboratory of Medicinal Chemical Biology, College of Pharmacy and Tianjin Key Laboratory of Molecular Drug Research, Nankai University, Haihe Education Park, 38 Tongyan Road, Tianjin, 300353, People’s Republic of China;

2 Thompson Rivers University, Manna, British Columbia

3 Tianjin University of Traditional Chinese Medicine, Tianjin, 300193, People’s Republic of China

* Corresponding author: Yuanyuan Hou, Gang Bai

1. **Synthesis section**

**1.1 General Chemical Reagents and Methods**

All purchased reagents for synthesis were used without further purification. All solvents were available commercially, dried or freshly dried and distilled before use. Thin-layer chromatography (TLC) was performed on silica gel GF254 plates with detection using short wave UV light (λ=254 nm) and staining with 10% phosphomolybdic acid in EtOH, followed by heating on a hotplate. Flash chromatography was performed with silica gel (100-200 mesh) with EtOAc/ petroleum ether or CH_2_Cl_2_/ MeOH as eluent. 1H and 13C NMR spectra were recorded on a Bruker AV 400 spectrometer at 400 MHz (^1^H NMR) and 101 MHz (^13^C NMR), using CDCl_3_ as solvents. Coupling constants are reported in Hertz.

**1.2 Synthesis of baicalein probe**

2 ml of DMF was added to a 15 ml round bottomed flask containing 27 mg of compound 1 (0.1 mmol, 1.0 EQ) and 12.8 mg of KHCO_3_. Dripped 24.8 mg iododiacridine (0.105 mmol, 16.4 μl, 1.05 eq) into the reaction system, and oil bath at 70 ℃ for 12 h. TLC was used to detect the reaction (dichloromethane: methanol = 20:1, RF = 0.4). The reaction mixture was quenched with 1 M HCl, 5 ml water and 5 ml ethyl acetate, and the organic phase was washed with saturated salt water for three times. Dried the product with anhydrous sodium sulfate and spin dry. The residue was purified by flash chromatography using DCM/MeOH 50:1 for elution. Then the baicalein probe (BP Probe 1) (12.1 mg, 0.059 mmol, 31% yield) was obtained. ^1^H NMR (400 MHz, Chloroform-d) δ 12.52 (s, 1H), 7.90 – 7.86 (m, 2H), 7.53 (d, J = 7.3 Hz, 4H), 6.68 (s, 1H), 6.56 (s, 1H), 4.00 (t, J = 6.2 Hz, 2H), 3.00 (s, 1H), 2.08 – 2.02 (m, 4H), 1.76 (t, J = 7.3 Hz, 2H). 13C NMR (101 MHz, Chloroform-d) δ 182.8, 164.4, 151.8, 150.7, 146.3, 132.0, 131.6, 129.3, 126.4, 106.5, 105.1, 91.6, 77.4, 69.6, 64.3, 32.7, 29.8, 29.5, 27.4, 13.4.

Figure S1. Synthetic route for baicalein probe.


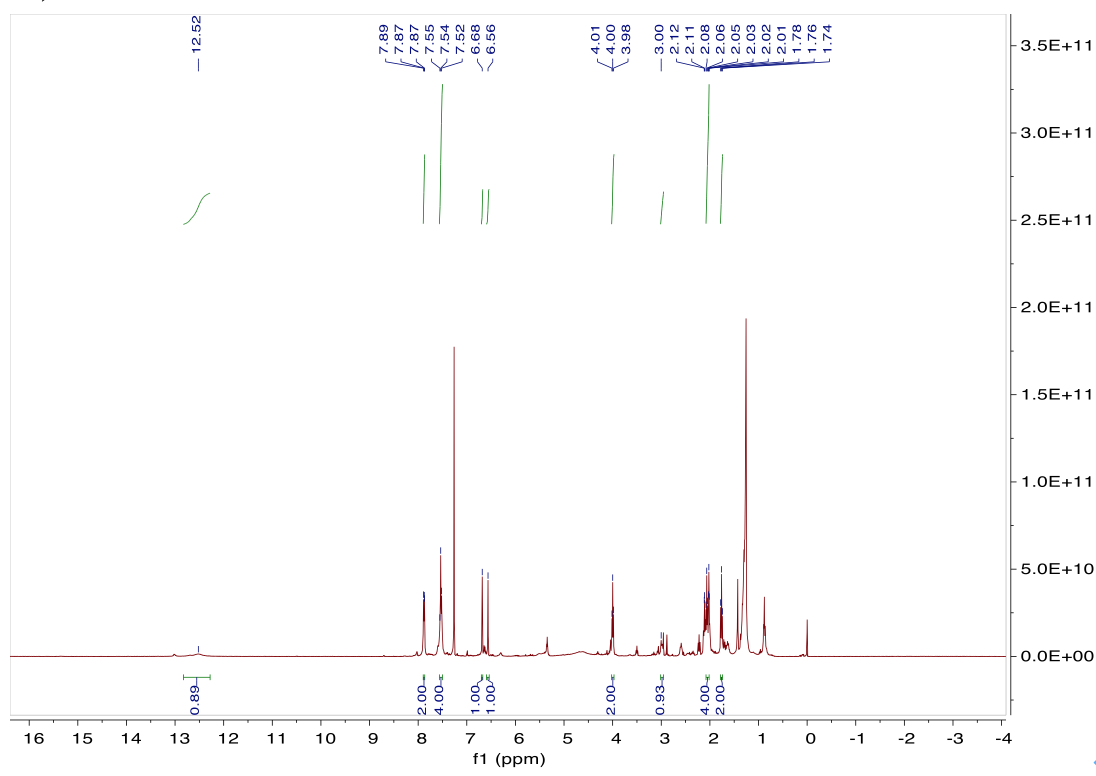


**A**


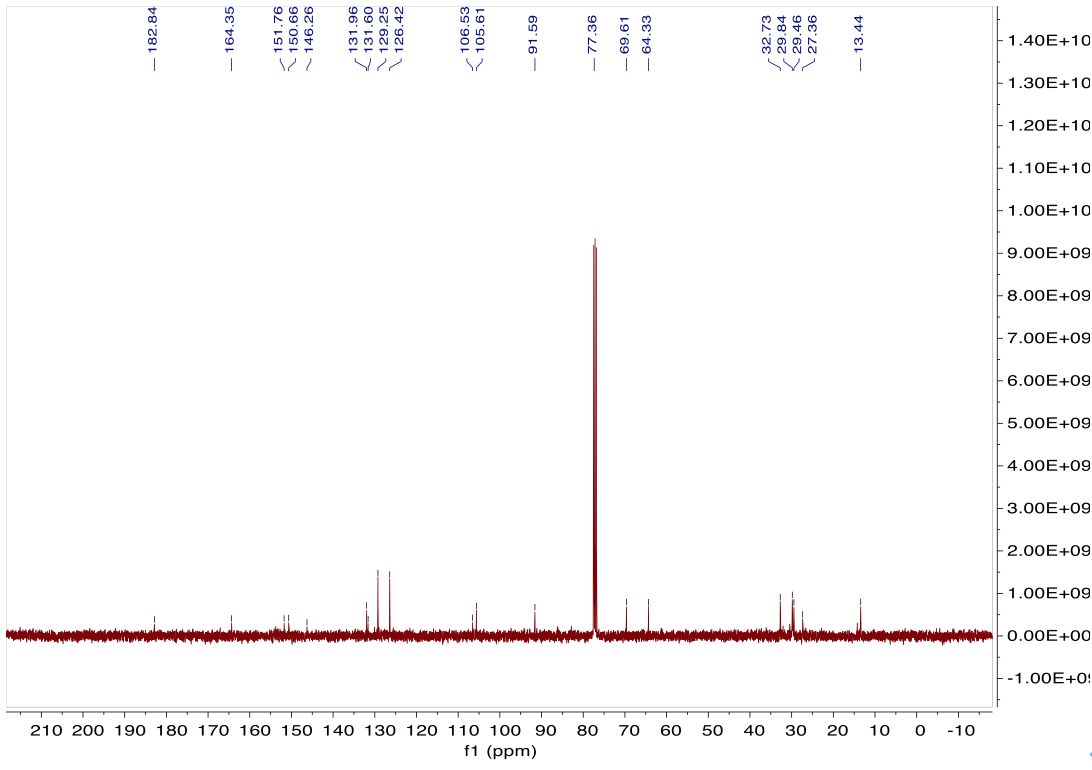


**B**

Figure S2. The NMR data of baicalein probe. (A) The ^1^H NMR data of baicalein probe. (B) The ^13^C NMR data of baicalein probe.

**1.3 Synthesis of alkynyl-baicalein**

Baicalein (54 mg, 0.2 mmol), KHCO3 (30 mg, 0.3 mmol) and DMF (2 ml) were added into a 50 ml single mouthed flask and stirred for 1 hour. Bromopropyne (25 mg, 16.4 UL, density: 1.52) was dripped and oil bath at 70 ℃ for 12 hours. TLC (DCM: MeOH = 15:1) was used to confirm that the raw material point disappeared and a new point was formed. 1 M HCl was added to quench the reaction, 5 ml water and 5 ml EA were added. The organic phase was washed three times with saturated salt water, dried with anhydrous sodium sulfate and then dried. 18.1 mg of the product was obtained from the coarse scraping plate (DCM: MeOH = 25:1), which was verified by NMR. ^1^H NMR (400 MHz, Chloroform-d) δ 12.54 (s, 1H), 7.89 (dd, J = 7.9, 1.8 Hz, 2H), 7.53 (d, J = 7.4 Hz, 3H), 6.75 (s, 1H), 6.69 (s, 1H), 5.29 (s, 1H), 4.90 (d, J = 2.5 Hz, 2H), 2.63 (t, J = 2.4 Hz, 1H). ^13^C NMR (101 MHz, Chloroform-d) δ 182.8, 164.457, 150.7, 150.4, 146.2, 132.0, 131. 5, 130.2, 129.2, 126.4, 106.6, 105.5, 92.3, 77.3, 77.2, 57.1.

Figure S3. Synthetic route for alkynyl-baicalein.

**A**

**B**

Figure S4. The NMR data of alkynyl-baicalein. (A) The ^1^H NMR data of alkynyl-baicalein. (B) The ^13^C NMR data of alkynyl-baicalein.

**1.4 Synthesis of baicalein probe-coumarin**

Alkynyl-baicalein (308 mg, 1 mmol) was added successively into 5 mL of a compound of tetrahydrofuran and water (1:1), then azide-coumarin (205 mg, 1.1 mmol) was slowly added into the mixture that was cooled with an ice bath. After then CuSO_4_.5H2O (500 mg, 2 mmol) and sodium ascorbate (396 mg, 2 mmol) were added the mixture, the ice bath was removed. After 12 hours, the TLC detection indicated that the reaction was completed. After that, the mixture was extracted with dichloromethane, and the aqueous phase was extracted twice with dichloromethane. The organic layers were combined and dried with anhydrous sodium sulfate, and were further concentrated to give crude product. Then the crude product was purified by column chromatography to give a pale-yellow solid. The high-resolution mass spectrometry [M+H]^+^ calculated 512.1088, found 512.1087, [M+Na]+ calculated 534.0908, found 534.0909.

Figure S5. Synthetic route for baicalein probe-coumarin.


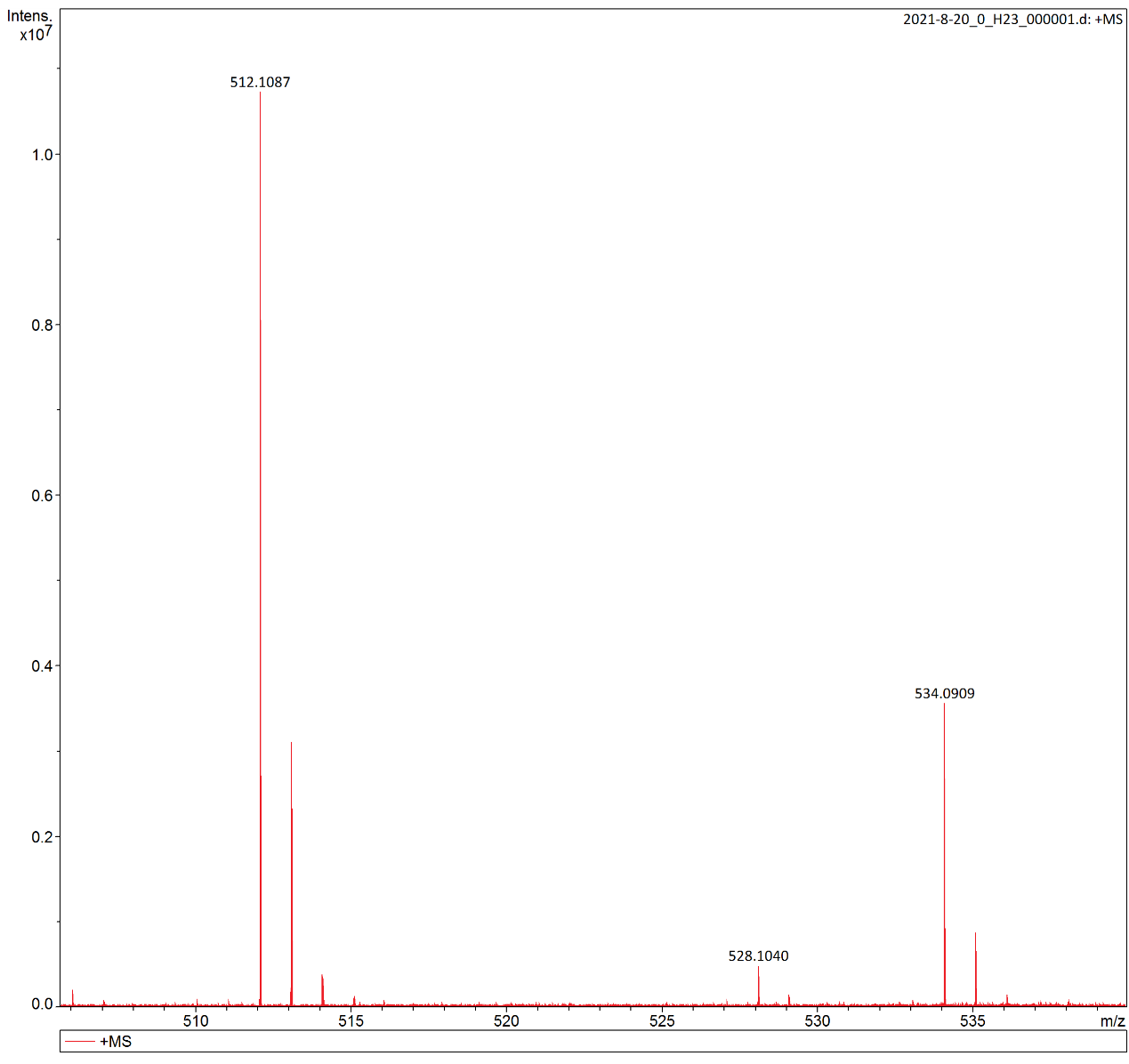


Figure S6. The high-resolution mass spectrometry of baicalein probe-coumarin.

**2. NO assay of baicalein probes**


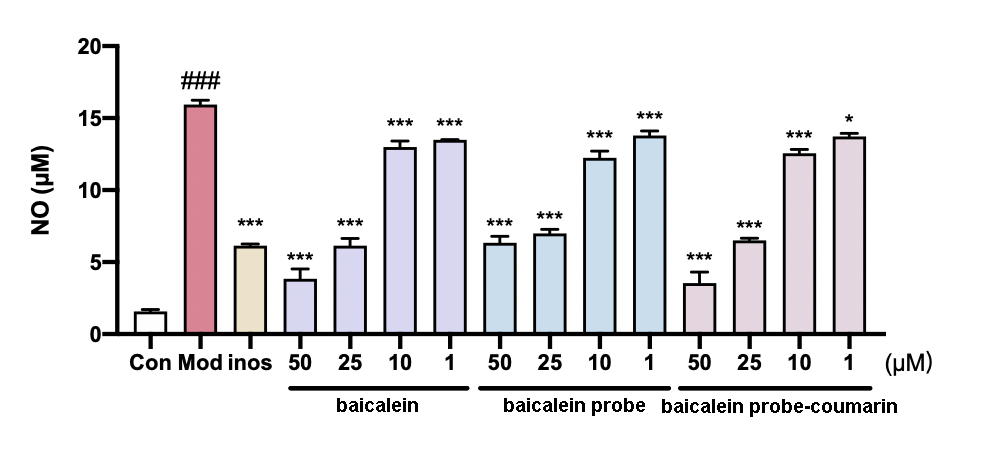


Figure S7. The effects of baicalein and baicalein probes on LPS-induced NO expression.

**3. Protein expression and purification**

The gene of wild-type (WT) HSP90 (residues 1-236) or its mutant (S52G, D93G, F138G, T184G) was cloned into the pET-28a expression vector, respectively. The recombinant plasmid was transformed into *E. coli* strain BL21 (DE3) and overexpressed as a 6 × His tag fused at the C terminus fusion protein. SDS-PAGE analysis revealed over 98% purity of the final purified recombinant protein. The purified protein was concentrated to 2.5 mg/mL.

**
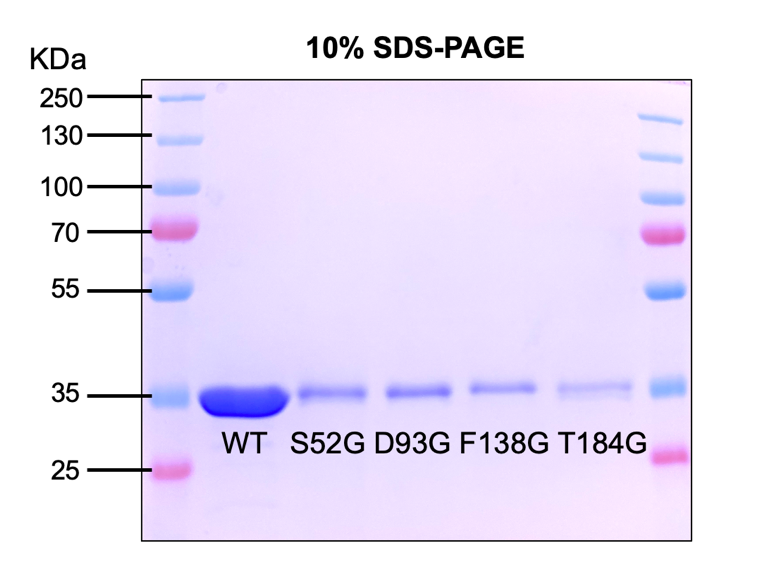
**

Figure S8. The Purification of wild-type or mutant HSP90.

**4. SPR analysis of baicalin and HSP90**

**
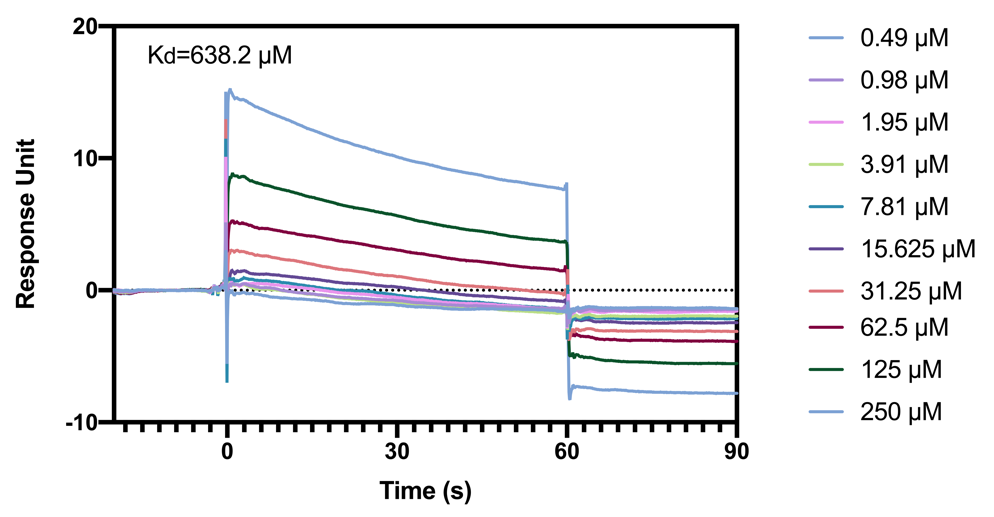
**

Figure S9. SPR analysis of interactions between baicalin and wild-type HSP90.

**5. FRET analysis**

HSP90 proteins working as a fluorescent donor with excitation and emission wavelengths were 290 nm and 332 nm, respectively. BP-coumarin was applied as a fluorescent receptor with excitation and emission wavelengths were 350 nm and 475 nm, ensuring the probability of FRET.

**
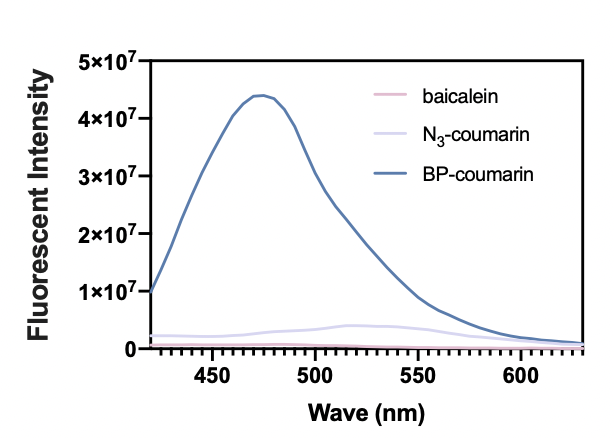
**

Figure S10. Fluorescence emission spectrum of BP-coumarin.

**6. SPR analysis of baicalein and mutant HSP90**

**
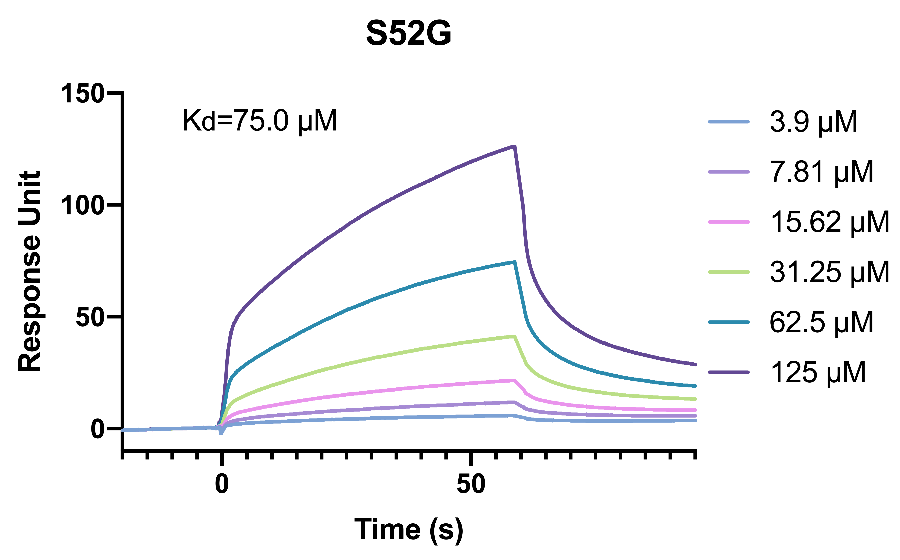
**

Figure S11. SPR analysis of interactions between baicalein and S52G mutant of HSP90.

**
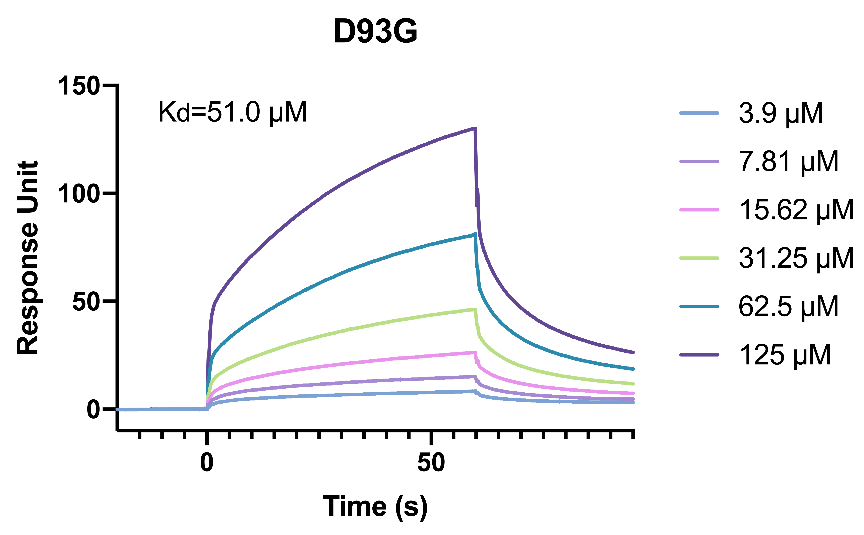
**

Figure S12. SPR analysis of interactions between baicalein and D93G mutant of HSP90.

**
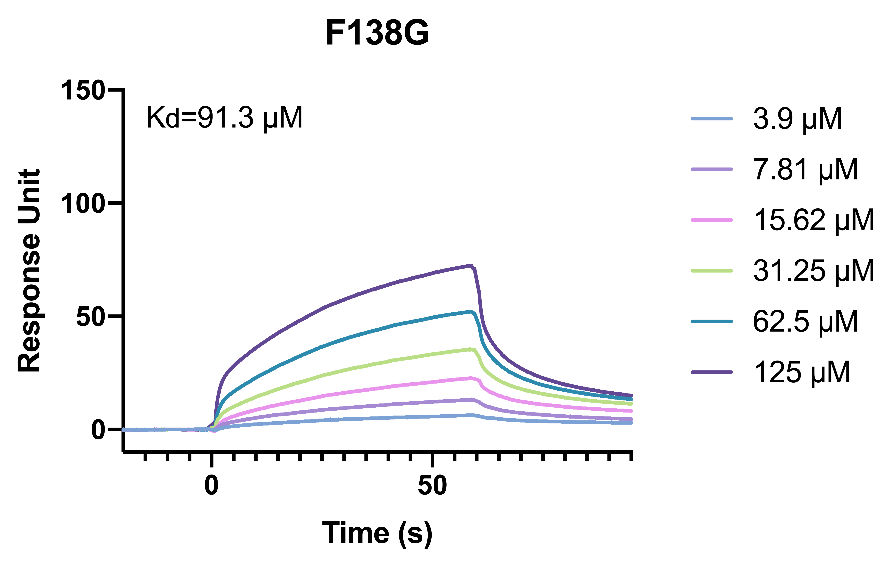
**

Figure S13. SPR analysis of interactions between baicalein and F138G mutant of HSP90.

**
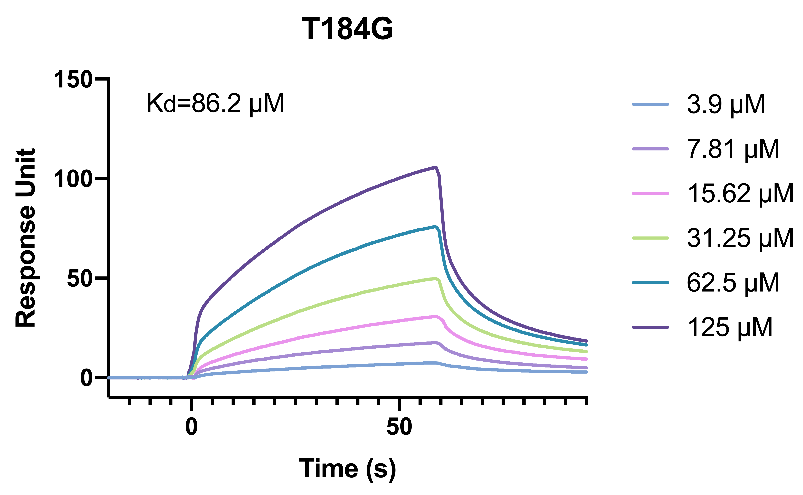
**

Figure S14. SPR analysis of interactions between baicalein and T184G mutant of HSP90.

**7. Sketch map for new client proteins discovery of HSP90**


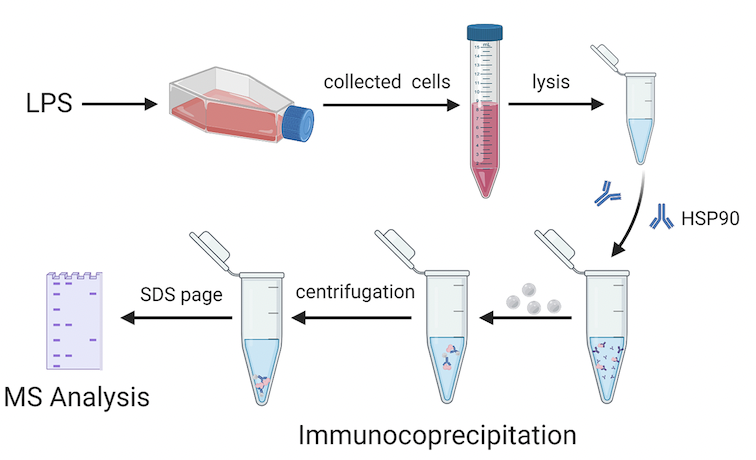


Figure S15. Experimental process of Co-IP.

**8. MST analysis of COX-2 and baicalein**


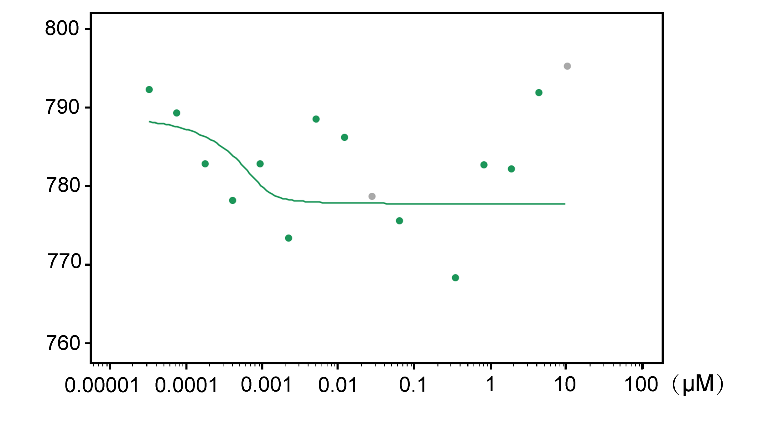


Figure S16. MST analysis of COX-2 and baicalein.

**9. Molecular dynamics simulation**


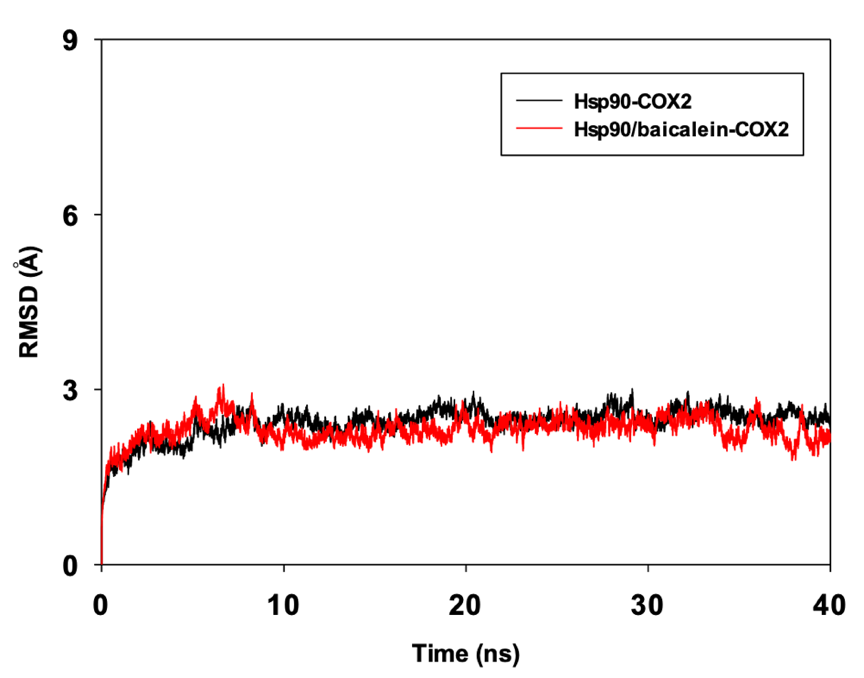


Figure S17. The root-mean-square deviations (RMSDs) of all the atoms of HSP90-COX-2 complex and HSP90/baicalein-COX-2 complex with respect to its initial structure as function of time.


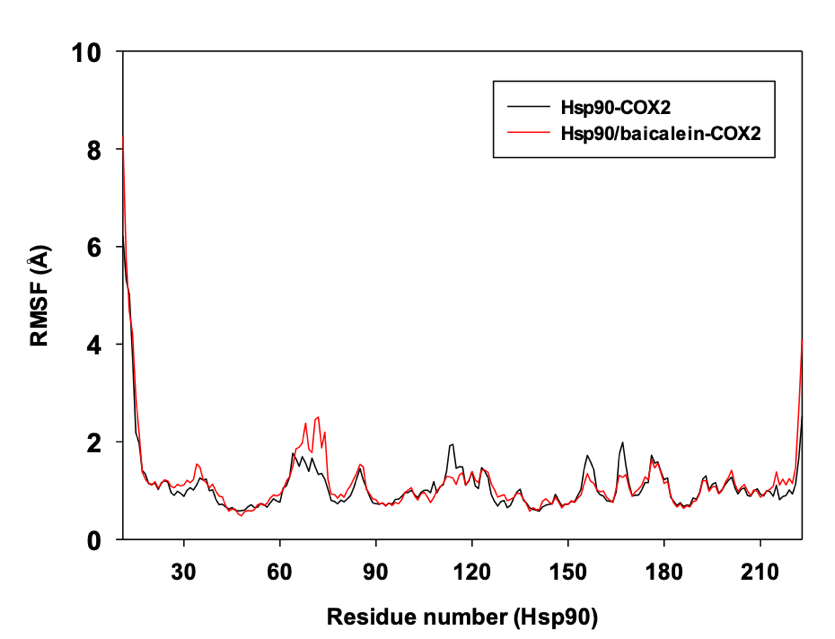


Figure S18. RMSF of residues of the HSP90 protein in HSP90-COX2 complex and HSP90/baicalein-COX2 complex during the 40 ns simulation.


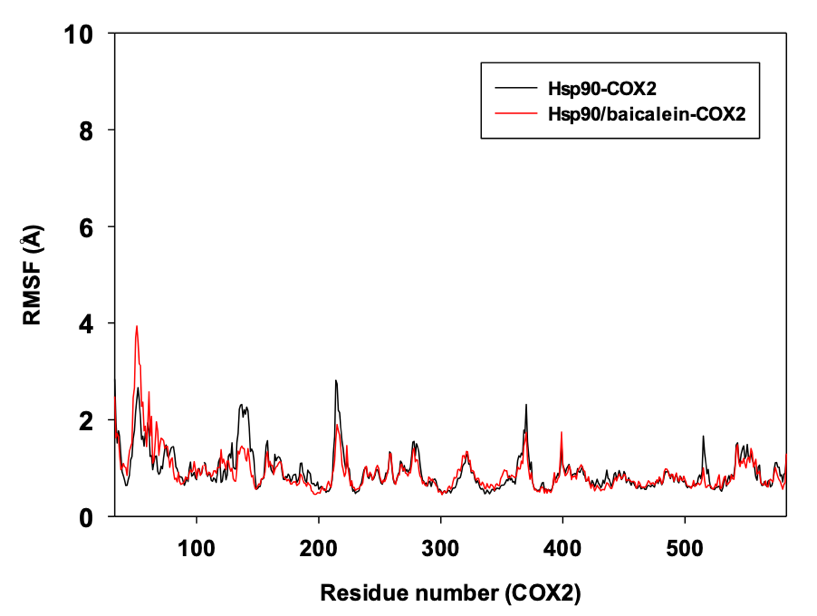


Figure S19. RMSF of residues of the COX-2 protein in HSP90-COX-2 complex and HSP90/baicalein-COX2 complex during the 40 ns simulation.

**10. Baicalein downregulated protein level of COX-2 stimulated by LPS.**


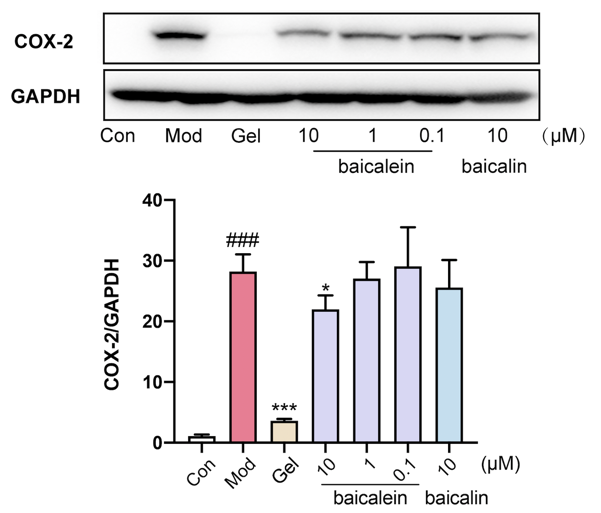


Figure S20. Baicalein downregulated the LPS-stimulated COX-2 protein expression. Cells were treated by LPS with or without drugs for 24 h. Bars represent the mean ± SD (n = 3). Geldanamycin (10 μM) was set as a positive control. ^###^P < 0.001 compared with the Con group; ^**^P < 0.01, ^***^P < 0.001 compared with the Mod group.

**11. The effect of baicalein on COX-2 and COX-1 under the pre-incubation of HSP 90α/β siRNA (m) stimulated by LPS**


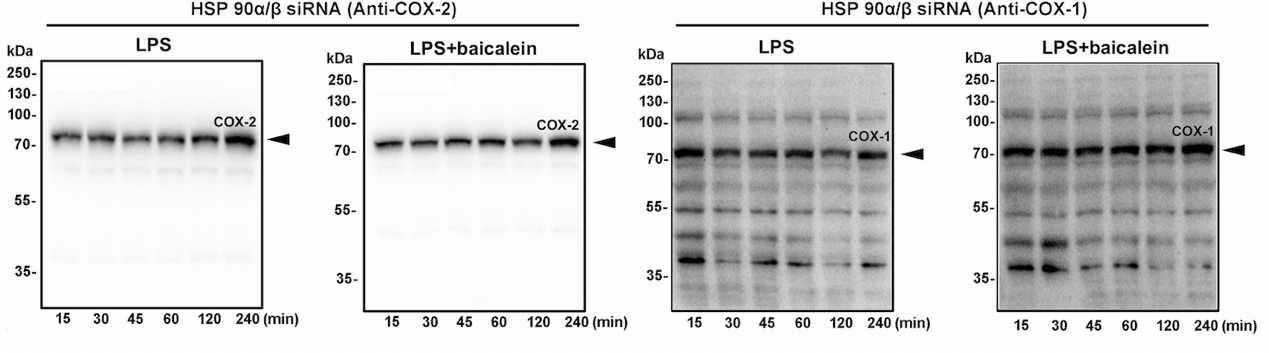


Figure S21. RAW264.7 cells were transfected with HSP 90α/β siRNA (m) for 20 h in advance and then stimulated by 100 ng/mL LPS with or without 10 μM baicalein.

**12. HSP90 ATPase domain inhibitors play antipyretic effect *in vivo***


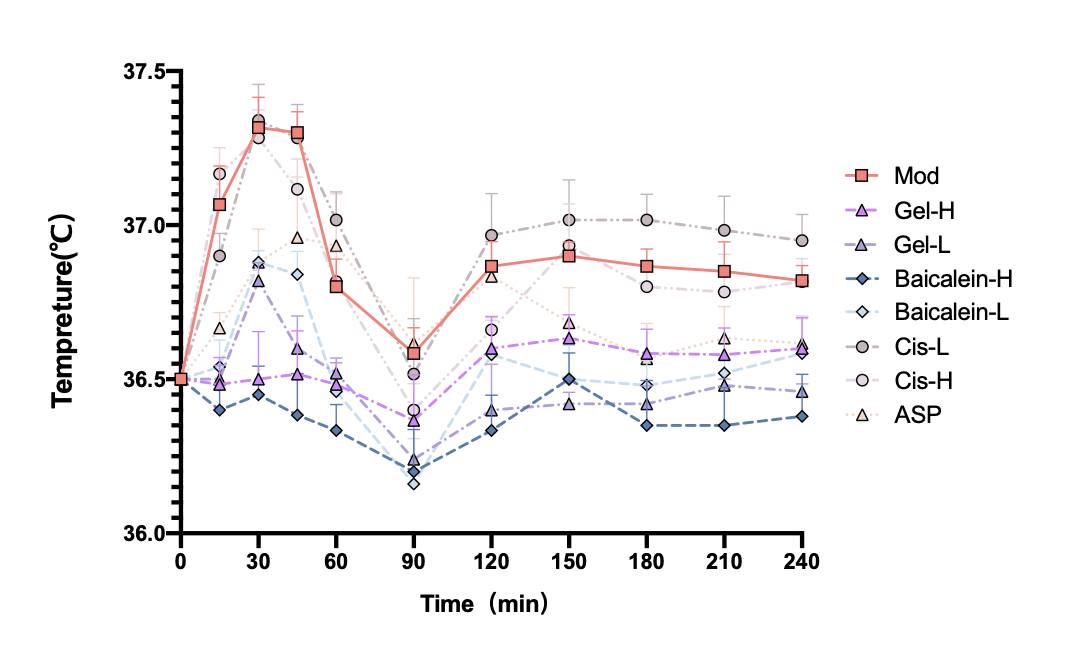


Figure S22. Comparison of the antipyretic activities of Gel, baicalein, Cis and ASP in rats.

**13. MG132 reduced the antipyretic effect of baicalein *in vivo***

**
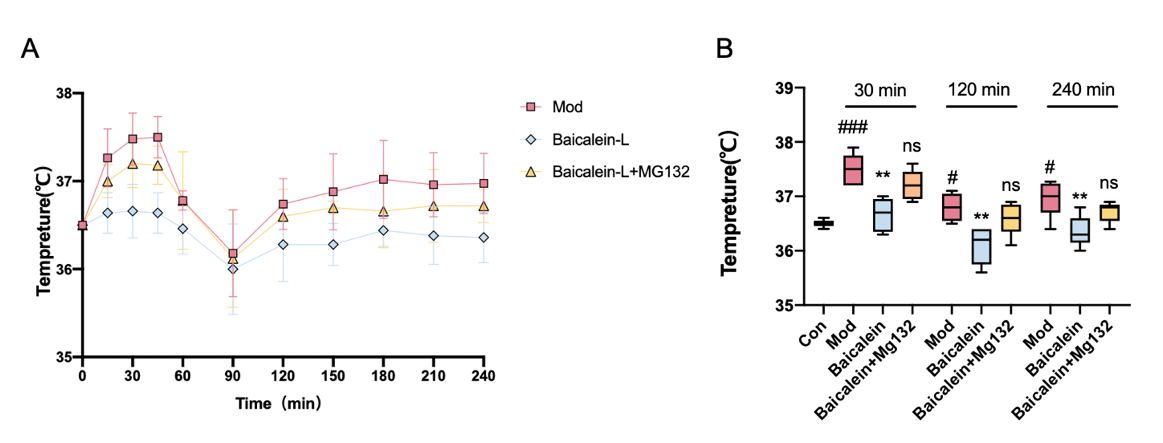
**

Figure S23. Comparison of the antipyretic activities of baicalein (1 mg/kg) and baicalein (1 mg/kg) plus MG132 (1 mg/kg).

**13. Protein profiling of Co-IP**

Table S1 Proteins captured by HSP90

| Gene Name | Mass (Da) | Score | Matches | Coverage |
| --- | --- | --- | --- | --- |
| Pkm | 57937 | 373 | 24(12) | 40% |
| Eef1a1 | 50141 | 228 | 16(10) | 23% |
| Vim | 53652 | 224 | 11(7) | 21% |
| Atp5f1b | 56560 | 218 | 14(8) | 24% |
| Hspd1 | 61055 | 147 | 7(6) | 13% |
| Cct3 | 60534 | 124 | 14(4) | 17% |
| Ptpn6 | 67561 | 73 | 4(2) | 7% |
| env | 68822 | 72 | 2(1) | 3% |
| P4hb | 57116 | 70 | 5(1) | 10% |
| Rpn2 | 69284 | 66 | 4(2) | 7% |
| Canx | 67278 | 63 | 4(2) | 6% |
| Cct4 | 58066 | 60 | 2(1) | 3% |
| Gdi2 | 50537 | 52 | 3(1) | 5% |
| Cct6a | 58044 | 49 | 4(1) | 7% |
| Ehd4 | 61481 | 47 | 3(1) | 5% |
| Pdia3 | 56678 | 47 | 4(2) | 8% |
| Rpn1 | 68528 | 44 | 5(2) | 10% |
| Ugt1a8 | 59742 | 43 | 4(1) | 4% |
| Ezr | 69413 | 42 | 6(1) | 6% |
| Syncrip | 69603 | 41 | 1(1) | 2% |
| **COX2** | **69013** | **34** | **2(2)** | **4%** |
| Mettl3 | 64474 | 36 | 7(0) | 3% |
| Spata7 | 67719 | 29 | 4(0) | 1% |
| Gpnmb | 63923 | 28 | 4(1) | 4% |
| Ces1d | 61788 | 25 | 1(1) | 2% |

**14. The predicted binding modality of the HSP90-COX-2 complex and HSP90/baicalein-COX-2 complex**

Table S2 Residues contributed to HSP90-COX-2 complex

| HSP90-COX-2 | | | | HSP90/baicalein-COX-2 | | | |
| --- | --- | --- | --- | --- | --- | --- | --- |
| Residues | Protein | Force | Values (kcal/mol) | Residues | Protein | Force | Values (kcal/mol) |
| Asp-57 | HSP90 | *∆E_ele_* | -35.0 | Asp-54 | HSP90 | *∆E_ele_* | -46.3 |
| Arg-60 |  |  | -25.4 | Asp-57 |  |  | -26.8 |
| Glu-62 |  |  | -11.8 | Arg-60 |  |  | -19.7 |
| Lys-209 |  |  | -9.9 |  |  |  |  |
| Met473 | COX-2 | *∆E_ele_* | -99.2 | Met-473 | COX-2 | *∆E_ele_* | -70.8 |
| Leu-480 |  |  | -36.5 | Leu-480 |  |  | -51.3 |
| Thr-70 |  |  | -8.4 |  |  |  |  |
| Arg-95 |  |  | -21.8 |  |  |  |  |
| Tyr-61  Met-130  Phe-213 | HSP90 | *∆E_vdw_* | <4.5 | Phe-213 | HSP90 | *∆E_vdw_* | <4.5 |
| Lys-82  Pro-92  Ser-96 | COX-2 |  |  | Leu-78  Lys-82 | COX-2 |  |  |
| total binding free energy | HSP90-COX-2 | *∆G_bind_* | -55.9 | total binding free energy | HSP90-COX-2 | *∆G_bind_* | -47.5 |
