# Supplementary material for: A novel mode of action for COX‐2 inhibition: Targeting ATPase domain of HSP90 induces ubiquitin degradation of new client protein COX‐2
Source: Clin Transl Med. 2022 Jan 24;12(1):e705. doi: 10.1002/ctm2.705 (PMC8787097; doi:10.1002/ctm2.705)
Supplement: Supplementary file 2 — Supporting Information [file CTM2-12-e705-s001.docx]

**Supporting Information 2:** Materials and methods

**A novel mode of action for COX-2 inhibition: baicalein targets the ATPase domain of HSP90, inducing ubiquitin degradation of its newly identified client protein COX-2**

Man Zhang^1^, Jing Cui^1^, Fukui Shen^1^, Lili Ye^1^, Chuanjing Cheng^1^, Yang Li^1^, Qiuyang Zhang^2^, Lin Niu^3^, Yuanyuan Hou^1^*, Gang Bai^1^*

1 State Key Laboratory of Medicinal Chemical Biology, College of Pharmacy and Tianjin Key Laboratory of Molecular Drug Research, Nankai University, Haihe Education Park, 38 Tongyan Road, Tianjin, 300353, People’s Republic of China;

2 Thompson Rivers University, Manna, British Columbia

3 Tianjin University of Traditional Chinese Medicine, Tianjin, 300193, People’s Republic of China

* Corresponding author: Yuanyuan Hou, Gang Bai

**1 METHODS**

**1.1 Cell lines and compounds**

RAW264.7 cells were purchased from CELLCOOK Biotechnology Co., Ltd. (Guangzhou, China). All cell culture reagents were purchased from Gibco BRL Life Technologies (NY, USA). Cells were cultured in the corresponding medium with 10% fetal bovine serum (FBS) at 37°C with 5% CO_2_. Aspirin (ASP) was purchased from Solarbio Co., Ltd. (Beijing, China). Geldanamycin (Gel), AT13387, cisplatin (Cis) and cucurbitacin D were purchased from Med Chem Express Co., Ltd. (NJ, USA). COX-2 proteins were purchased from Sino Biological (Beijing, China). ATP, baicalein, baicalin, paracetamol, ibuprofen, indometacin and mefenamic acid were obtained from Shanghaiyuanye Bio-Technology (Shanghai, China). Lipopolysaccharide (LPS), HSP 90α/β siRNA (m) were purchased from Santa Cruz Technology (TX, USA). Primary antibodies (Rabbit) against HSP90, COX-1, COX-2, AKT, JNK, P-AKT^473^, P-JNK and GAPDH, and secondary antibodies (goat anti-rabbit IgG H&L) used for western blotting were purchased from Cell Signaling Technology (MA, USA). Alexa Fluor® 594 antibody (goat anti-rabbit IgG H&L) used for the colocalization of HSP90 proteins and baicalein was purchased from Abcam (Cambridge, UK). COX-2 primary antibody (Mouse) and goat anti-mouse IgG (H&L) secondary antibody (Alexa Fluor® 647) used for the colocalization of HSP90 proteins and COX-2 proteins were purchased from Santa Cruz Technology (TX, USA) and Thermo Fisher (MA, USA), respectively.

**1.2 Heat-clearing experiments in Rats**

Male SD rats were obtained from Beijing Vital River Laboratory Animal Technology Co., Ltd. Thirty rats were divided into six groups (5 rats per group): The Mod group (LPS 40 μg/kg), the ASP group (LPS 40 μg/kg + ASP 100 mg/kg), the three baicalein groups (LPS 40 μg/kg + baicalein 15 mg/kg, 50 mg/kg or 150 mg/kg) and the baicalin group (40 μg/kg LPS + baicalin 150 mg/kg). Rats were intraperitoneally injected with LPS and drugs were immediately administered by intragastric gavage. The anal temperature of rats was measured via a BL-420 biological function experiment system (Chengdu Taimeng Software Co., Ltd.) once every 15 min for an hour prior to drug administration and once every 30 min over the next 3 h. Another forty-five rats were divided to nine groups (5 rats per group): The Con group, Mod group (LPS 40 μg/kg), Gel groups (LPS 40 μg/kg + Gel 10 mg/kg or 1 mg/kg), baicalein groups (LPS 40 μg/kg + baicalein 10 mg/kg, or 1 mg/kg), Cis groups (40 μg/kg LPS + Cis 10 mg/kg or 1 mg/kg) and ASP group (LPS 40 μg/kg + ASP 20 mg/kg). Rats were intraperitoneally injected with both LPS and drugs simultaneously. Then the blood of rats was obtained to detect the protein levels of COX-2 and COX-1. The anal temperature of rats was also measured. All animal experiments were conducted following the National Institutes of Health Guide for the Care and Use of Laboratory Animals (NIH Publications No. 8023, revised 1978) (TCM-LAEC2016031).

**1.3 Nitric oxide (NO) assay**

RAW264.7 cells were cultured in RPMI-1640 standard growth medium (4.5 g/L glucose, L-glutamine, sodium pyruvate) with 10% FBS and 1% penicillin/streptomycin. When cells achieved approximately 80%, cells were plated in 96-well plates in RPMI-1640 standard growth medium without FBS and cultured for 24 h. Then 1 μg/mL LPS with or without baicalein, baicalin or baicalein probe were added to cells for 24 h. 50 μL supernatant of cells were collected to detect the nitric oxide content in different groups using NO kit (Beyotime Biotechnology, Shanghai, China).

**1.4 Synthesis of baicalein probes (baicalein probe and baicalein probe-coumarin)**

For capturing and tracing the target proteins of baicalein, baicalein probes including the baicalein probe and baicalein probe-coumarin were synthesized. The phenolic hydroxyl position of the C-14 site of baicalein was modified, baicalein probe has been introduced with an alkynyl photosensitive label. baicalein probe-coumarin was synthesized with alkynyl-baicalein and azido-coumarin tag. The detailed synthesis methods and data are shown in the supporting information 1 (Figure S1-S6).

**1.5 Target capturing and analysis**

RAW264.7 cells were plated in 75 cm^2^ petri dishes until they reached approximately 80% confluence. LPS (100 ng/mL) with 1 μM baicalein probe (the target-capturing group) or 1 μM baicalein probe combined with 5 μM baicalein (the competition group) was added to the cells for 24 h, respectively. The cells were placed in a UV cross-linker, the ultraviolet wavelength was 365 nm, and the cells were irradiated for 30 min. After photosensitive catalytic crosslinking, the cells were washed 3 times with precooled phosphate buffer saline (PBS). Then, 600 μL RIPA lysis buffer was added to the cells and the mixture was kept on ice for 30 min. The lysates were collected, centrifuged and used for the next operation. One milliliter of NH_2_-magnetic microspheres (MMs) (5 mg/mL) was reacted with sulfo-SADP (0.5 mg, 11 μmol) in 5 mL borate buffer (pH 9) to obtain azide-modified MMs. Then the above protein solution containing baicalein probe was connected to azide-modified MMs, which product named BP-MMs, by a click reaction at 4°C for 12 h, according to the previous reports.^1^ A magnet was used to separate the BP-MMs, and 500 μL DL-dithiothreitol (DTT; 100 μM) was added to the BP-MMs at 4°C for 30 min to release the target proteins. Coomassie Brilliant Blue staining and protein profiling (Huada Protein R & D Center Co., Ltd, Beijing) were utilized to detect the captured target proteins.

**1.6 Colocalization of HSP90 proteins with baicalein probe-coumarin**

RAW264.7 cells were seeded in glass-bottom dishes at approximately 60% confluence, 10 μM baicalein probe-coumarin tag was added to the cells to reveal the location of baicalein with pseudo green fluorescence. After washing with PBS, 5% goat serum was added for blocking the nonspecific binding. A rabbit anti-HSP90 antibody (1:200) was added to the cells overnight at 4°C. A secondary antibody (Alexa Fluor® 594) was used to visualize the location of HSP90 proteins with pseudo red fluorescence. All images were obtained by a laser confocal microscope (Leica TCS SP8).

**1.7 Western blot**

The protein levels of HSP90, COX-2, P-AKT^473^, AKT, P-JNK, JNK, and GAPDH were analyzed by western blotting. After drug treatment, cell lysates were obtained using radioimmunoprecipitation assay (RIPA) lysis buffer containing 1% protein phosphatase inhibitor (P1260, Solarbio, Beijing) and 1% phenylmethanesulfonyl fluoride (P0100, Solarbio, Beijing). Then, the cell lysates were centrifuged and used for protein quantification by a BCA kit (Solarbio, Beijing). 15% SDS-PAGE gels were used to separate the proteins. Then, the proteins were transferred to a PVDF membrane for blocking and incubation with primary and secondary antibodies. Blots were captured by an automatic chemiluminescence imaging analysis system (Tanon-5200) and analyzed by ImageJ software.

**1.8 Surface plasmon resonance (SPR) analysis**

Wild-type (WT) and mutant HSP90 recombinant protein were expressed and purified in *Escherichia coli* system. The details of the expression and purification process were described in the supporting information (Figure S7). A Biacore T200 optical biosensor (GE Healthcare, Pittsburgh, PA, USA) was used to perform SPR experiments. Sodium acetate buffer (pH 5.5) immobilized WT or mutant HSP90 proteins (100 μg/mL). In the competitive test, ATP (0.156 mM to 2.5 mM) were set up with or without 40 μM baicalein diluted in the PBS buffer. During each binding cycle, molecules over a range of concentrations were injected at a flow rate of 30 μL/min for 1 min, and the dissociation was monitored for 300 s. The data were collected and organized according to the previous research.^2^

**1.9 Fluorescence Resonance Energy Transfer (FRET)**

The FRET assay was according to the previous research with an appropriately modification.^3^ HSP90 proteins working as a fluorescent doner with excitation and emission wavelengths were 290 nm and 332 nm. Baicalein probe-coumarin was applied as a fluorescent receptor with excitation and emission wavelengths were 350 nm and 475 nm (Figure S8). The FRET was performed on a Hitachi fluorescence spectrophotometer (F-4500, Japan). Baicalein probe-coumarin and Gel were dissolved in DMSO to form 10 mM solutions. HSP90 proteins were transferred to PBS assay buffer before use. Each quartz cell contained 10 μM baicalein probe-coumarin and 50 μM HSP90 proteins in a final volume of 1 mL. For the competition, 50 μM Gel was added to HSP90 proteins before baicalein probe-coumarin was incubated. Baicalein probe-coumarin, HSP90 proteins, or Gel alone were set up as control solutions.

**1.10 Fluorescence-based thermal shift (FTS) assay**

The wild-type HSP90 proteins and baicalein were diluted in PBS. Baicalein was complexed with HSP90 protein (10 μM) at a 10:1 ratio in 96-well plates. Then, Protein Thermal Shift Dye Kit^TM^ (1:6,000) was added to the wells to give a total volume of 20 μL. A real-time PCR machine (LightCycler 96, Roche, Switzerland) was used to scan the fluorescence intensity of each well at 0.03°C/s from 37°C to 55°C.

**1.11 Circular dichroism (CD) spectroscopy**

HSP90 protein was dialyzed against sodium phosphate buffer (pH 7.4). 40 μM HSP90 proteins with 10 μM baicalein were incubated at 4°C for 6 h. The same amount of baicalein and HSP90 proteins were set as a control group. A MOS-450 spectropolarimeter (Bio-Logic, France) equipped with a Peltier unit was used for CD spectral analysis at 20°C.

**1.12 Coimmunoprecipitation (Co-IP)**

RAW264.7 cells were grown in 100 mm cell culture dishes with LPS (100 ng/mL) stimulation for 24 h. Baicalein (10 μM) was added to the cell lysates for 12 h at 4°C. Subsequently, 1 μL HSP90 or COX-2 antibody was added to 50 μL cell lysate. The immune complexes were incubated in a four-dimensional spinner at 4°C for 8 h. Then, 20 μL protein G agarose beads (37478S, Cell Signaling Technology, USA) were added and incubated in a shaker at 4°C for 3 h. The beads were precipitated by centrifugation and washed with precooled PBS. Then, the beads were boiled with SDS sample buffer for 5 min at 100°C for western blotting.

**1.13 Molecular docking and molecular dynamics (MD) simulations**

Molecular docking was performed to investigate the binding mode between HSP90 and COX-2 using the ZDOCK server (zdock.umassmed.edu). The 3D structures of HSP90 (PDB ID: 1YET) and COX-2 (PDB ID: 1PXX) were downloaded from the RCSB Protein Data Bank (www.rcsb.org). For docking, the default parameters were used as described in the ZDOCK server. As judged by the docking score, the top-ranked configuration was subjected to an MD study to revise the docking result. The MD simulations were performed on a Dell Precision T5500 workstation according to the previous research.^4^

**1.14 Binding free energy and energy decomposition per residue calculations**

The Molecular Mechanics/Generalized Born Surface Area (MM/GBSA) was used to calculate the binding free energies (*ΔG*_bind_ in kcal/mol). To identify the key protein residues responsible for the ligand-binding process, the binding free energy was decomposed on a per-residue basis.^5^ For each complex, the binding free energy of MM/GBSA was estimated as follows:

*ΔG*_bind_= G_complex_ ‒ G_protein_ ‒ G_ligand_

where *ΔG*_bind_ is the binding free energy and G_complex_, G_protein_ and G_ligand_ are the free energies of the complex, protein, and ligand, respectively.^6^

**1.15 Negative stain electron microscopy**

HSP90 proteins, COX-2 proteins and the combination of the two proteins with or without baicalein were dissolved in PBS. Uranium acetate was used to dye the samples. The samples were air-dried at room temperature and a 120 kV transmission electron microscope (TEM; Talos F200C, FEI, USA) was utilized to observe the morphology of the proteins.

**1.16 Statistical analysis**

Prism 9 (GraphPad Software, San Diego, GA) was utilized to perform statistical analysis. Significance was accepted at the P < 0.05 level. Significant differences between two groups were assessed using *t*-tests, and analysis of multiple groups was performed using one-way ANOVA. The results are shown as the mean ± SD in the histogram. In the boxplot, bars represent medians with 25th and 75th percentiles made using GraphPad Prism 9. Comparison between the groups was performed using Mann–Whitney U test.

**REFERENCES**

1. Fang R, Cui Q, Sun J, et al. PDK1/Akt/PDE4D axis identified as a target for asthma remedy synergistic with beta2 AR agonists by a natural agent arctigenin. *Allergy.* 2015;70:1622-32.

2. Fu X, Wang Z, Li L, et al. Novel Chemical Ligands to Ebola Virus and Marburg Virus Nucleoproteins Identified by Combining Affinity Mass Spectrometry and Metabolomics Approaches. *Sci Rep.* 2016;6:29680.

3. Kim J, Felts S, Llauger L, et al. Development of a fluorescence polarization assay for the molecular chaperone Hsp90. *J Biomol Screen.* 2004;9:375-81.

4. Zhang A, Yue Y, Yang Y, et al. Discovery of N-(4-fluoro-2-(phenylamino)phenyl)-pyrazole-4-carboxamides as potential succinate dehydrogenase inhibitors. *Pestic Biochem Physiol.* 2019;158:175-84.

5. Liu H, An X, Li S, et al. Interaction mechanism exploration of R-bicalutamide/S-1 with WT/W741L AR using molecular dynamics simulations. Mol Biosyst. 2015;11:3347-3354.

6. Bai LS, Xu JJ, Zhao CX, et al. Enhanced hydrolysis of beta-cypermethrin caused by deletions in the glycin-rich region of carboxylesterase 001G from Helicoverpa armigera. *Pest Manag Sci.* 2020;77:2129-2141.
